# Wearing Glasses

**Published:** 1876-09

**Authors:** 


### WEARING GLASSES.

The age at which it becomes necessary to wear glasses depends so much upon the habits of the individual, and the care that has been taken of the eyes, that no rule, in this respect, can be established. Many of the ills of life come through ignorance of, or indifference to, the plainest teachings of reason and experience.

The eye is the most sensitive and delicate organ in the body, and the one which is, perhaps, subjected to the greatest abuse. The loss of sight, through neglect or ignorance, deprives a great calamity of all consolation. There is, then, no duty more imperative than to protect the eyes against whatever might injure them, and to aid them at the proper time by the best and most approved appliances of optical science. An effort to save money by purchasing poor and cheap glasses, will, in time, prove to be the most unfortunate extravagance.

When the eye is in a natural and healthy condition, we find that the sight becomes impaired, at some time, between the age of thirty-five and fifty, depending upon the care that has been observed in using the eyes. Whenever we are obliged to hold a book or newspaper eighteen or twenty inches from the eyes in order to see the letters distinctly, the aid of glasses is indicated. Now, it is a very easy matter to purchase a pair of some itinerant oculist, that will magnify the letters and enable us to read them ; but it is a very imprudent thing to do, and it may result in total loss of vision.

The sale of cheap spectacles should be punished by law, just as other frauds and impositions are punished. If any one will take the trouble to carefully examine the cheap glasses—they really cost no less than good ones—that are largely sold throughout the country, he will not wonder that cases of impaired sight and blindness are becoming alarmingly frequent.

When examined with a magnifying glass of medium power, such glasses present a curious appearance. They seem to be opaque, and have a gray and turbid appearance, as if milk and particles of dirt had been mingled in the material from which they were made. The wearing of such glasses must strain the eyes and cause impairment of sight, beside rendering the wearer liable to cataract and other diseases of the eye.

Whenever glasses fatigue the eyes, and cause them to smart and become watery, one of two conditions is pretty certain to exist : either the glasses are not adapted to the present requirements of the eye, or else they are defective. In either case they are certain to do permanent injury to the eyes if their use is long persisted in.

The best glasses, like all things good and pure, can bear the closest inspection. When examined with a magnifying glass they are found to be perfectly transparent. If the least flaw or imperfection is discovered, they are cast aside. In this age of competition, no respectable manufacturer would risk his reputation by sending out inferior goods. Beside, experienced opticians know better than others the injury that might result from the use of imperfect glasses ; and no honorable man would resort to such deception.

As a rule, "the best is always the cheapest" in the long run. Persons who require glasses should ascertain the exact distance that it is necessary to hold a page of Hall's Journal of Health from the eyes in order to distinctly read it without glasses, and if there is no reliable optician near, the order, giving the distance in inches, should be sent to some well-known manufacturer of spectacles in one of the large cities, and he can easily select a pair of glasses that will suit. A satisfactory and reliable article can in this way be procured at about the cost of the worse than worthless variety usually sold by peddlers. When we speak of the best glasses we do not refer to the Brazilian pebbles. They are greatly inferior to glasses that are now produced by the best makers. The principal objection to Brazilian pebbles is that they polarize light, a quality most injurious to the eyes. The finest eye glasses, with rubber, steel, or celluloid frame, cost from the manufacturer about $2.50 a pair. Best spectacles, with solid fine gold frame, about five dollars a pair.

